# Fern mycorrhizae do not respond to fertilization in a tropical montane forest

**DOI:** 10.1002/pei3.10139

**Published:** 2024-03-29

**Authors:** Thais Guillen, Michael Kessler, Jürgen Homeier

**Affiliations:** ^1^ Department of Systematic and Evolutionary Botany University of Zurich Zurich Switzerland; ^2^ Faculty of Resource Management University of Applied Sciences and Arts (HAWK) Göttingen Germany

**Keywords:** AMF, Ecuador, ferns, fertilization, mycorrhizae, nitrogen, phosphorus

## Abstract

Ferns are known to have a lower incidence of mycorrhization than angiosperms. It has been suggested that this results from carbon being more limiting to fern growth than nutrient availability, but this assertion has not been tested yet. In the present study, we took advantage of a fertilization experiment with nitrogen and phosphorus on cloud forest plots of the Ecuadorean Andes for 15 years. A previous analysis revealed changes in the abundances of fern species in the fertilized plots compared to the control plots and hypothesized that this might be related to the responses of the mycorrhizal relationships to nutrient availability. We revisited the plots to assess the root‐associated fungal communities of two epiphytic and two terrestrial fern species that showed shifts in abundance. We sampled and analyzed the roots of 125 individuals following a metabarcoding approach. We recovered 1382 fungal ASVs, with a dominance of members of Tremellales (Basidiomycota) and Heliotales (Ascomycota). The fungal diversity was highly partitioned with little overlap between individuals. We found marked differences between terrestrial and epiphytic species, with the latter fundamentally missing arbuscular mycorrhizal fungi (AMF). We found no effect of fertilization on the diversity or relative abundance of the fungal assemblages. Still, we observed a direct impact of phosphorus fertilization on its concentration in the fern leaves. We conclude that fern–fungi relationships in the study site are not restricted by nutrient availability and suggest the existence of little specificity on the fungal partners relative to the host fern species.

## Graphical abstract

In the present study, we assessed the root‐associated fungi of two epiphytic and two terrestrial fern species in a 15‐year fertilization experiment in the Ecuadorean Andes. We found no effect of fertilization on the diversity or abundance of the fungal assemblages associated with ferns.

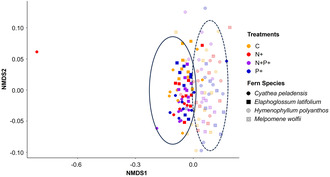

## 1 INTRODUCTION

The symbiosis of land plants and mycorrhizal fungi was instrumental in the evolution of terrestrial ecosystems and remains essential for most vascular plants (Brundrett & Tedersoo, 2018; Strullu‐Derrien et al., 2018). Arbuscular mycorrhizal fungi (AMF, phylum Glomeromycota), in particular, form the most common type of mycorrhizae (Brundrett, 2004; Öpik et al., 2006). They grow their hyphae within the plant roots and often improve the host's water uptake and access to limiting soil nutrients, such as phosphorus and nitrogen, among other benefits (Brundrett, 2002, 2009; Morgan & Connolly, 2013). Most research regarding AMF has focused on angiosperms and economically important crop species (Ganugi et al., 2019). However, little is known about the ecology and functionality of mycorrhizal associations in ancient plant groups such as ferns. Ferns played a crucial ecosystem role before the rise of angiosperms to ecological dominance around 100 mya ago in the Angiosperm Terrestrial Revolution (ATR) (Benton et al., 2022). Today, they are the second‐most diverse lineage of land plants with about 11.500 species (PPG I, 2016). Their diversity and abundance are concentrated in humid environments, particularly tropical mountains (Kessler et al., 2011; Suissa et al., 2021; Weigand et al., 2020).

Few studies have considered the relationship between mycorrhizal fungi and ferns. For a long time, these studies relied solely on visual inspection of roots, which prevented fungal identification (e.g., Field et al., 2015; Kovács et al., 2007; Rimington et al., 2015; West et al., 2009). They documented the presence of AMF and dark septate endophytes (DSE) in fern roots (Lehnert et al., 2017). DSE are mainly members of the phylum Ascomycota, and they are often found associated with ferns when AMF are not present (Lehnert et al., 2017; Muthuraja et al., 2014). These findings suggest a switch from AMF to DSE as allies during fern evolution (Lehnert et al., 2017), but until now there are no studies exploring fern‐DSE interactions. During the last decade, molecular methods have shed new light into the composition of fungal communities associated with fern (Cook & Taylor, 2023, Guillen‐Otero et al., 2023, 2024; Perez‐Lamarque et al., 2022; Sandoz et al., 2020; Strullu‐Derrien et al., 2018). Based on our current state of knowledge, ferns have a lower incidence of mycorrhizal colonization than angiosperms, with about 34% of all fern species sampled so far lacking mycorrhizae, as opposed to about 18% in angiosperms (Lehnert et al., 2017; Wang & Qiu, 2006). This can be linked in part to the high proportion of ferns growing epiphytically (about 27% versus about 9% in angiosperms; Zotz et al., 2021) since overall mycorrhizal fungi are soil‐bound (Brundrett & Tedersoo, 2018; Willis et al., 2013). Mycorrhizae are also largely absent in fern species associated with swampy habitats, because arbuscular mycorrhizal fungi cannot tolerate the anoxic conditions of inundated soils (Helgason & Fitter, 2009; Mellado‐Mansilla et al., 2022; Tedersoo, 2017). Still, even among terrestrial ferns growing in forests, a considerable proportion of species has few or no AMF partners (Lehnert et al., 2017). It has been suggested that the causes of this phenomenon might be the existence of a greater limitation in development by assimilate availability than by nutrient availability, compared to angiosperms (Kessler et al., 2010, 2014). However, except for a greenhouse experiment on a single fern species (Guillen‐Otero et al., 2024), to date there are no experimental studies manipulating nutrient levels to assess the importance of soil nutrients in determining mycorrhization in ferns.

The Nutrient Manipulation Experiment (NUMEX) is a long‐term nutrient manipulation experiment carried out at the Ecuadorean Andes, where plots in natural montane cloud forest have been fertilized with nitrogen and phosphorus since 2008 (Homeier et al., 2012; Velescu et al., 2021). Previously, Weigand et al. (2022) found systematic variations in the abundances of fern species in the fertilized plots compared to the control ones, with some species increasing in abundance while others decreased. The authors proposed that these differential responses are related to the taxonomic affiliations of the species and their ecological strategies. Thus, species belonging to ancient evolutionary families that originated in a nutrient‐poor world before the ATR may be more strongly dependent on mycorrhizae than modern fern families which evolved after the ATR under more nutrient‐rich conditions (Benton et al., 2022; Lehnert et al., 2017). Furthermore, Weigand et al. (2022) suggested that fern species displaying a conservative life strategy in the epiphytic habitat are more severely affected due to reduced competitive ability, decreased drought tolerance, or modifications of the mycorrhizal relationships. However, the authors did not assess the mycorrhizal associations of the 153 species recorded in their study, so that the effects of fertilization on the root‐associated fungi remain unknown. Finally, given that AMF usually improve the absorption of phosphorus (P) and nitrogen (N) by their host (Brundrett, 2009; Morgan & Connolly, 2013), it could be possible to determine the response of fern–fungi relationships to fertilization by analyzing the concentration of these nutrients in fern leaves.

In the present study, we revisited the parcels examined by Weigand et al. (2022). We used a metabarcoding approach to identify the root‐associated fungi in 125 individuals of four fern species that previously showed significant shifts in their abundances. The species selected included two terrestrial and two epiphytic species, one each belonging to phylogenetically old and modern families, respectively. In this way, our sampling covered the variability of phylogenetic associations and growth habits proposed by Weigand et al. (2022) as influential on the responses of fern mycorrhizae to fertilization. Three of the study species had shown decreases related to fertilization, whereas one had increased (Weigand et al., 2022). Our basic hypothesis was that fertilization would lead to systematic shifts in the relative abundance and composition of fungal communities particularly AMF communities in the studied fern species. More specifically, we expected (i) fertilization to lead to a decrease in the intensity of mycorrhization, since nutrients would be less limiting, (ii) phosphorus addition to show stronger effects than nitrogen addition, in accordance with previous studies of tree‐associated mycorrhizae in the same study plots (Duenas et al., 2020) and with the generally held notion that mycorrhizae are particularly important for the uptake of phosphorus (Brundrett, 2009), and (iii) fertilization to increase leaf nutrient concentrations.

## 2 MATERIALS AND METHODS

### 2.1 Study site

We conducted our study at the Reserva Biológica San Francisco (RBSF) (03°58'S, 79°4'W), which is located to the north of Podocarpus National Park in Zamora‐Chinchipe, Ecuador. The climate of the study area is tropical perhumid, with a mean annual temperature of about 14.5°C and mean annual precipitation of about 2000 mm and least rainfall from November to March (Bendix et al., 2011; Wolf et al., 2011). Soils are stagnic cambisols (Homeier et al., 2012). Soils are heterogeneous but generally nutrient‐poor compared to other tropical montane forests, with low N and P concentrations (Wolf et al., 2011). Data based on the monitoring of bulk and dry deposition between 1998 and 2012 indicate natural annual depositions of 47 ± 20 kg N ha^−1^, as well as 2.4 ± 4.3 kg P ha^−1^ (Homeier et al., 2012; Wilcke et al., 2013, 2019). The forest at the study site has a canopy height of 18–22 m.

### 2.2 The nutrient manipulation experiment (NUMEX)

The NUMEX experiment has been continuously running since 2008 (Homeier et al., 2012). On a broad ridge at 2020–2120 m a.s.l., 16 permanent plots of 400 m^2^ each (20 m × 20 m) were established in a stratified random design, arranged in four blocks each made up of four plots, namely an unfertilized control and the three plots with additions of N, P, and N+P. Plots in each block were at least 10 m apart. To minimize the chance of horizontal nutrient transfer by water movement through the soil, the plots were positioned in a way that small natural obstacles (ridges, trenches) lie between them, with the control preferably put at the highest terrain. The nutrients have been added twice per year in the following concentrations, representing a moderate nutrient addition: 50 kg ha^−1^ year^−1^ of N (as urea CH_4_N_2_O), 10 kg ha^−1^ year^−1^ of P (as NaH_2_PO_4_), or a combination of both, with the granulate dispersed directly to the forest floor. For further details see Homeier et al. (2012) and Velescu et al. (2021).

### 2.3 Study species and field sampling

We selected four fern species based on the results of Weigand et al. (2022), using the following criteria: (i) the species should be present in each plot, (ii) two species should be terrestrial (ground‐rooting) and two epiphytic, (iii) one terrestrial and epiphytic species each should belong to a phylogenetically old family, whereas the other should belong to the polypod radiation, and (iv) the species should have shown shifts in abundances, suggesting an effect of fertilization. These criteria quickly narrowed down the choice to four species. *Cyathea peladensis* (Hieron.) Domin (Cyatheaceae) is a small, terrestrial tree fern with trunks about 8–12 cm in diameter and up to 2 m tall and leaves 1–1.5 m long. It occurs in humid mid‐elevation forests from southern Colombia to Peru and is especially common on nutrient‐poor soils in ridge forest. The second terrestrial species was *Elaphoglossum latifolium* (Sw.) J.Sm. (Dryopteridaceae), a herbaceous fern species with short‐creeping rhizomes and undivided leaves typically 20–40 cm long. The taxonomy of this and closely related species remains unclear, but as treated here it occurs in humid montane forests from Mexico to Peru. It can grow both epiphytically and terrestrially, but at our study site is almost exclusively ground rooting. This was the only of the four study species that had increased in abundance in the fertilization plots in the study of Weigand et al. (2022). The first epiphytic species was *Hymnophyllum polyanthos* (Sw.) Sw. (Hymenophyllaceae), a small species with long‐creeping rhizomes and thin (1‐cell thick) leaves 10–20 cm long. It is a common species in cloud forests from across the globe. The final species was *Melpomene wolfii* (Hieron.) A.R.Sm. & R.C.Moran (Polypodiaceae), a purely epiphytic species with short‐creeping rhizomes and leaves 10–20 cm long. It occurs in cloud forests from Venezuela to Peru.

On May 27, 2022, we selected two individuals of each study species per plot, ideally located at a considerable distance within the plot to minimize the possibility of colonization by the same fungi. We collected about 10–15 root fragments (5 cm) from each plant, trying to harm the individuals as little as possible. However, in the case of small individuals (especially in *M. wolfii*), we had to collect the whole plant. The roots were placed in tea bags, rapidly dried in silica gel, and stored in air‐tight containers at room temperature. For the nutrient analyses, we sampled two mature leaves (in the case of *C. peladensis* two pinnae) by specimen and dried them in an oven at 60°C. The dried samples were stored in paper bags at room temperature. Because not all species were present with two individuals in each plot, the final number of samples was 125.

### 2.4 Leaf nutrient analysis

We grinded and homogenized the dried leaf samples and determined total organic carbon (C) and nitrogen (N) concentrations with a C/N elemental analyzer (Vario EL III, Hanau). To obtain the phosphorus (P) concentration, the material was digested with 65% HNO_3_ at 195°C for 8 h and then assessed using the ICP‐OES technique (inductively coupled plasma optical emission spectrometry, iCAP 7000, Thermo Fisher Scientific).

### 2.5 Molecular analysis

DNA extraction and purification were carried out following the metabarcoding protocol described by Guillen‐Otero et al. (2023) for root‐associated fungi in ferns and lycophytes. To recover an optimal representation of the general fungal community and the arbuscular mycorrhizal fungi (AMF) community we targeted the ITS rRNA region with the universal primers ITS1F (Gardes & Bruns, 1993) and ITS4 (White et al., 1990). This metabarcoding approach allowed us to gain a wider perspective of fern‐associated fungal communities and also served as a measure of the intensity of mycorrhization by relating the number of sequences of arbuscular mycorrhizal fungi to that of overall fungi (Guillen‐Otero et al., 2023, 2024). A sequencing approach targeting exclusively Glomeromycota with primers NS31 (Simon et al., 1992) and AML2 (Lee et al., 2008), for example, would likely have resulted in higher resolution for these fungi (Öpik et al., 2010); however, it would have failed in recovering the dominant fungal groups, and the intensity of mycorrhization would not have been be estimated.

In brief, approximately 50 mg of roots from each plant were used to perform the extraction with DNeasy Plant Mini Kit (Qiagen). We purified the resulting samples with Monarch Genomic DNA Purification Kit (New England Biolabs, Frankfurt am Main). The amplification and sequencing steps were performed by EzBiome. The genetic material was amplified using primers that contained Illumina adapter overhang nucleotide sequences: ITS1F (5' TCGTCGGCAGC GTCAGATGTGTATAAGAGACAG‐CTTGGTCATTTAGAGGAAGTAA)/ITS4 (5' GTCTCGTGGGCTCGGAGATGTGTATAAGAGA‐CAGTCCTCCGCTTATGATATGC). The resultant fragments were 450–550 bp in length. Finally, the obtained libraries were sequenced (2 × 300 bp paired‐end read setting) on the MiSeq (Illumina). Three samples (6C2, 13C2, 18 M2) failed the quality control analysis and therefore were excluded from further analyses.

Demultiplexed paired‐end reads were processed using the package dada2 (Callahan et al., 2016; v1.22.0) and its tutorial, in R (v4.1.3). In a nutshell, we confirmed the presence of primers and adaptors and removed them from the sequencing reads using Cutadapt (Martin, 2011; v4.3). We performed a filtering step considering the sequence quality and discarding those sequences with expected errors greater than 2 and length under 450 bases. We used a minimum overlap of 4 bases and a maximum mismatch of 2 bases to dereplicate and merge the paired reads and built an amplicon sequence variant (ASV) table. We utilized the Naïve bayesian classifier (RDP classifier) with the curated fungal reference sequences from UNITE (Abarenkov et al., 2024; v8.3) to identify the ASVs obtained and exclude those sequences that remained unidentified at the phylum level. For taxonomic nomenclature, we followed the classification of the kingdom fungi proposed by Wijayawardene et al. (2022). We excluded taxa with relative abundances lower than 1% in each sample to obtain the core microbiome by specimen as described by Guillen‐Otero et al. (2023). A table containing taxonomic classification and ASVs relative abundance for the general core microbiome dataset can be found in Appendix S1.

### 2.6 Statistical analyses

Statistical analyses were carried out in R (R Core Team, 2021; v4.1.2) and Microsoft Excel (v2305). To assess the representativeness of our sample size we plotted rarefaction curves using the function ggrare (mahendra‐mariadassou/phyloseq‐extended package; v 0.0.0.9000) for the general core microbiome.

In order to quantify the contribution of the corresponding treatment and host species to the general and arbuscular mycorrhizal fungal communities, we performed an additive diversity partitioning analysis (*β* = *γ* – *α*), where gamma diversity (*γ*) is the cumulative diversity within each fern species, alpha diversity (*α*) is the average diversity per specimen, and beta‐diversity (*β*) is the diversity among samples by treatment (Veech et al., 2002). We calculated and represented the values of *γ*, *α*_1_, *α*_2_, *β*_2_, and *β*_2_ by fern species using Microsoft Excel, where *γ* = fungal species total, *α*_1_ = fungal species average per fern species, *α*_2_ = fungal species average per treatment, *β*_1_ = *α*_2_ – *α*_1_, and *β*_2_ = *γ* – *α*_2_. This analysis allows a visual and quantitative representation of the overlap (resp. uniqueness) of the fungi between individuals, species, and treatments, and hence the importance of these factors in determining the fungal assemblages.

The relative compositions of the fungal communities at the order level and of arbuscular mycorrhizal fungal (AMF) communities at the family level were summarized with the ggplot package (v 3.4.2). To detect significant differences among treatments within each fern species, regarding fungal species richness (sum of ASVs in a sample) and relative abundance (number of sequences in a sample relative to all fungal sequences), leaf phosphorus concentration, and leaf nitrogen concentration, we performed an analysis of variance (ANOVA) by plant species. The data were previously tested for normality (Shapiro–Wilk test) and for homogeneity of variances (Bartlett's test).

Additionally, we carried out a PERMANOVA (permutate multivariate analysis of variance) test to assess the role of the applied treatments and the host on the fungal community composition, including an interaction factor. We used the adonis function (vegan package; v.2.6.4), with Bray–Curtis dissimilarity.

Finally, to visualize the dissimilarities in fungal community composition within fern species considering the influence of the applied treatment and plant species, we utilized a non‐metric multidimensional scaling (NMDS) analysis with Bray–Curtis as distance measure. Next, an indicator species analysis allowed us to identify the taxa associated with each of the conditions established on the treatments. The statistical test carried out 9999 permutations using the r.g function in the indicspecies package (De Caceres et al., 2016; v.1.7.12).

## 3 RESULTS

We recovered 10,181 fungal sequences in the 125 fern individuals sampled. After data cleaning and restricting the analysis to the core microbiomes, i.e., only those species representing at least 1% of the sequences in a given sample, the sequences were assigned to 1389 ASVs. Although the number of sequences was quite low in certain samples, the number of ASVs generally reached an asymptote in each sample, indicating sufficient sampling coverage (Figure S1).

**FIGURE 1:**
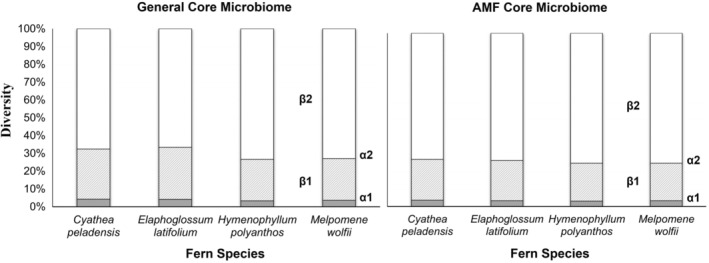
Additive diversity partitioning of the number of ASVs of root‐associated fungi in the general core microbiomes (left) and AMF (Glomeromycota) core microbiomes (right) in four species of ferns (*Cyathea peladensis* (Hieron.) Domin, *Elaphoglossum latifolium* (Sw.) J.Sm., *Hymnophyllum polyanthos* (Sw.) Sw., and *Melpomene wolfii* (Hieron.) A.R.Sm. & R.C.Moran). *α*_1_: average number of ASVs per individual, *α*_2_: average number of ASVs per nutrient treatment, *β*_1_: increase of the number of ASVs due to the inclusion of more individuals per treatment, *β*_2_: increase due to the inclusion of more individuals in different treatments.

The additive partitioning approach showed that, on average, each fern individual contained 4% of the total number of ASVs recorded in that species (*α*_1_ = 15 species) (Figure 1). This value is close to the number expected for 30–32 individuals per species if each had a totally unique fungal assemblage (3.1%–3.3%), indicating that there was very little overlap in fungi between fern individuals. This effect translated to the treatments (*α*_2_), with an overall average of 25%, the value expected if there is no overlap. In fact, even the ASV shared by the highest number of fern individuals was only recovered in 35 out of 125 individuals (28%), while 1223 ASVs (88.5%) were only found in a single fern specimen.

**FIGURE 2:**
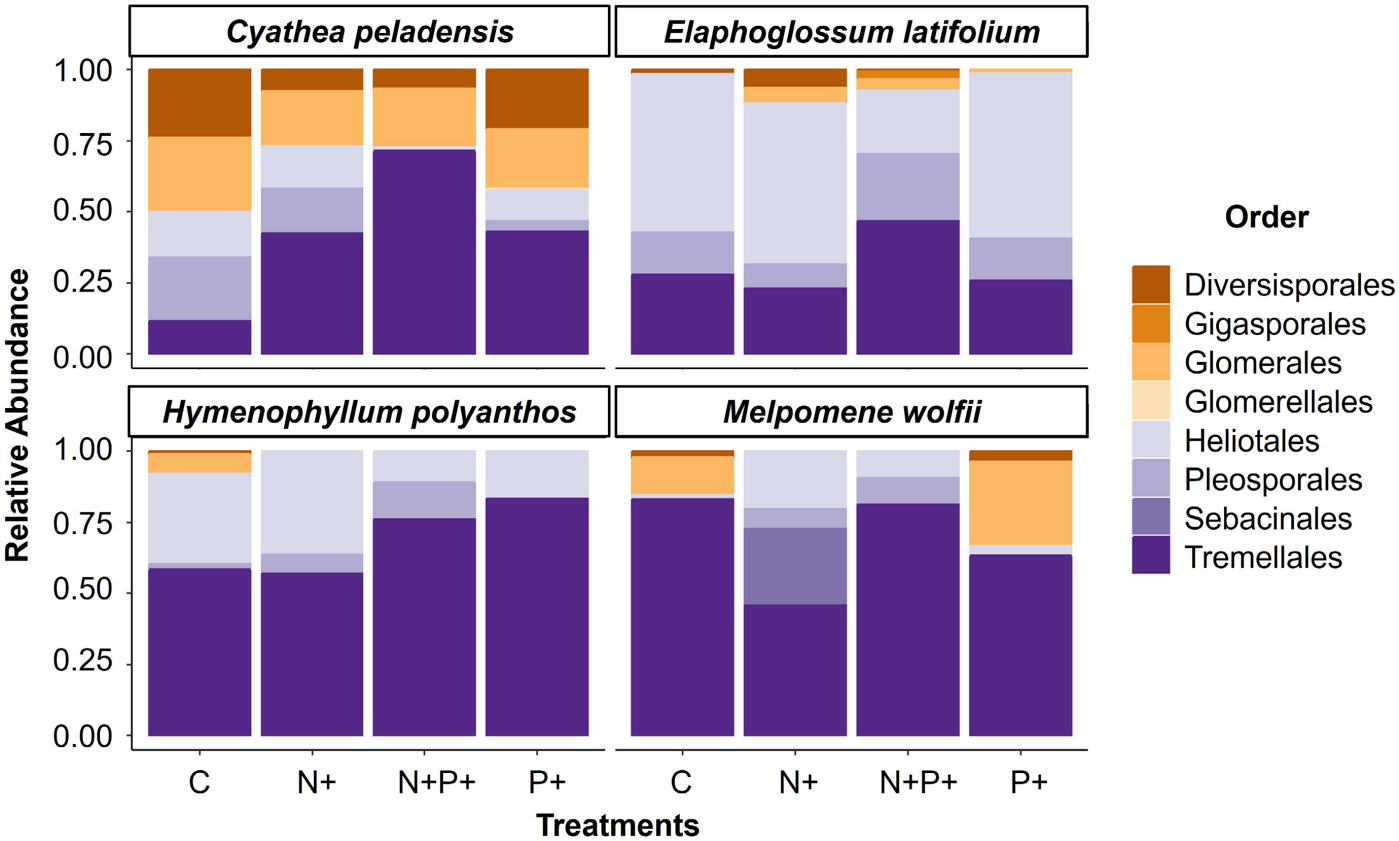
Relative abundances (based on numbers of sequences) of root‐associated fungi in four fern species (*Cyathea peladensis* (Hieron.) Domin, *Elaphoglossum latifolium* (Sw.) J.Sm., *Hymnophyllum polyanthos* (Sw.) Sw., and *Melpomene wolfii* (Hieron.) A.R.Sm. & R.C.Moran) in relation to four nutrient treatments. The taxonomic composition is represented at the order level. Values are averages of six to usually eight individuals per species and treatment.

**FIGURE 3:**
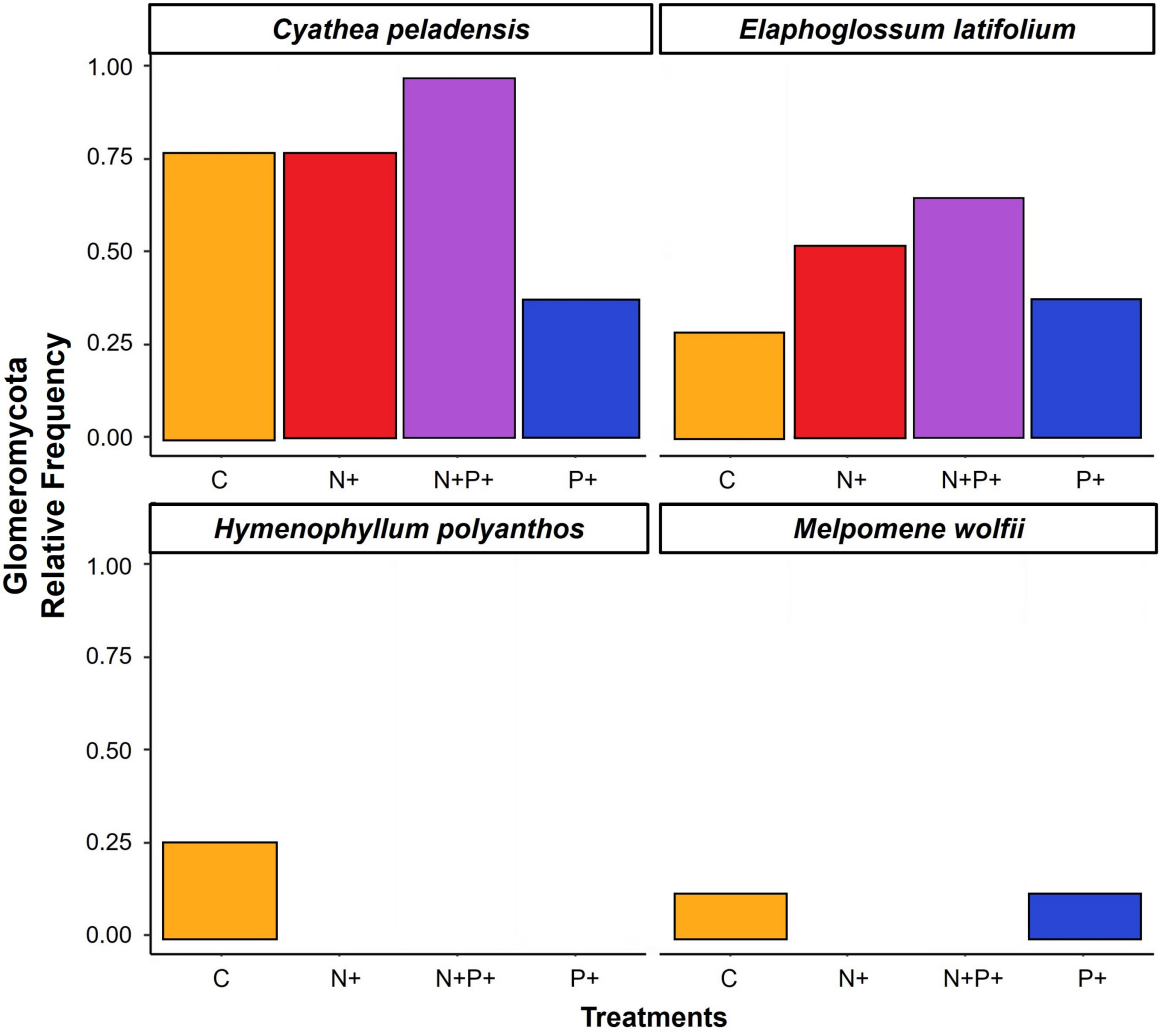
Relative frequency of root‐associated arbuscular mycorrhizal fungi (phylum Glomeromycota) in four fern species (*Cyathea peladensis* (Hieron.) Domin, *Elaphoglossum latifolium* (Sw.) J.Sm., *Hymnophyllum polyanthos* (Sw.) Sw., and *Melpomene wolfii* (Hieron.) A.R.Sm. & R.C.Moran) related to four nutrient treatments. Values are averages of six to usually eight individuals per species and treatment.

Taxonomic assignment by phylum associated 1055 (76%) of the ASVs to Ascomycota, 165 (11.9%) to Basiodiomycota, 89 (6.4%) to Rozellomycota, 75 (5.3%) to Glomeromycota, and the remainder to Chytridiomycota, Basidiobolomycota, and Mortierellomycota (0.4%). These relative abundances of ASVs overall were also reflected by the relative abundances by order in the four fern species. In general, we found a predominance of order Tremellales (Basidiomycota) and Heliotales (Ascomycota) both in the terrestrial and the epiphytic ferns (Figure 2). Focusing only on Glomeromycota (arbuscular mycorrhizal fungi), the sequences were identified as mainly belonging to the families Acaulosporaceae (35, 44.3%) and Glomeraceae (33 ASVs, 41.8%), with fewer Gigasporaceae (3 ASVs, 3.8%) and 8 ASVs (10.1%) unassigned. Glomeromycota were mainly restricted to the terrestrial fern species, with only scattered records among the epiphytic species (Figure 2; Figure 3). As for the overall fungal assemblages, we found no significant differences in the composition of the arbuscular mycorrhizal fungi (AMF) families relative to the nutrient treatment (Table 1). The indicator species analysis did not reveal significant results in terms of the AMF species that characterized each treatment by fern species.

**TABLE 1:** ANOVA results by fern species, using Bray–Curtis dissimilarities as distance measure and comparing the variation in fungal communities composition, leaf phosphorus concentration, and leaf nitrogen concentration per treatment.

		df	SS	MS	*F*	*p*
Cyathea peladensis						
Fungal species richness	Treatment	3	102.5	34.16	1.828	>.05
	Residuals	26	485.8	18.69		
Fungal relative abundance	Treatment	3	53.4	17.79	0.457	>.05
	Residuals	26	1011.2	38.89		
Leaf phosphorus concentration	Treatment	3	17.59	5.864	6.303	<.01**
	Residuals	26	24.19	0.930		
Leaf nitrogen concentration	Treatment	3	94.1	31.37	1.893	>.05
	Residuals	26	430.9	16.57		
Elaphoglossum latifolium						
Fungal species richness	Treatment	3	35.8	11.95	0.412	>.05
	Residuals	28	812.9	29.03		
Fungal relative abundance	Treatment	3	4.55	1.517	0.401	>.05
	Residuals	28	105.89	3.782		
Leaf phosphorus concentration	Treatment	3	18.993	19.66	6.331	<.001***
	Residuals	28	9.019	0.322		
Leaf nitrogen concentration	Treatment	3	22.4	7.467	0.516	>.05
	Residuals	28	404.9	14.462		
Hymenophyllum polyanthos						
Fungal species richness	Treatment	3	71.3	23.78	0.681	>.05
	Residuals	28	978.1	34.93		
Fungal relative abundance	Treatment	3	0.2942	0.09808	0.934	>.05
	Residuals	28	2.9389	0.10496		
Leaf phosphorus concentration	Treatment	3	2.083	12.82	0.694	<.001***
	Residuals	28	1.516	0.0542		
Leaf nitrogen concentration	Treatment	3	22.09	7.362	2.115	>.05
	Residuals	28	99.62	3.558		
Melpomene wolfii						
Fungal species richness	Treatment	3	69.6	23.19	0.691	>.05
	Residuals	27	906.6	33.58		
Fungal relative abundance	Treatment	3	0.3349	0.1116	0.954	>.05
	Residuals	27	3.1599	0.1170		
Leaf phosphorus concentration	Treatment	3	1.155	0.3851	4.036	<.05*
	Residuals	23	2.195	0.0954		
Leaf nitrogen concentration	Treatment	3	128.5	42.83	7.976	<.001***
	Residuals	26	139.6	5.37		

*Note*: Asterisks indicate the levels of statistical significance.

Abbreviations: df, degrees of freedom; *F*, *F* value; MS, mean of squares; SS, sum of squares.

**FIGURE 4:**
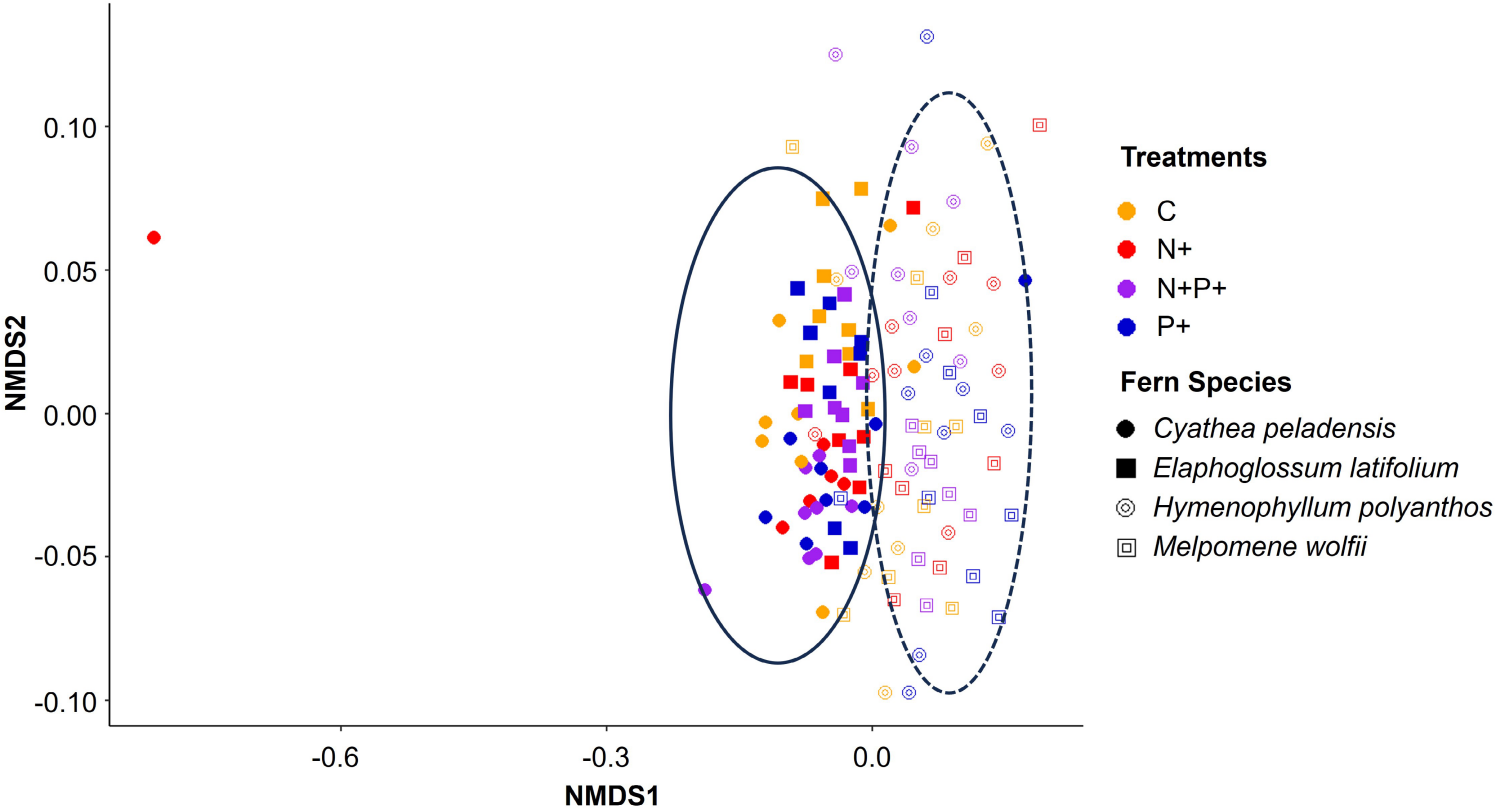
NMDS ordination of the root‐associated fungal assemblages associated to four fern species in four nutrient treatments. This graph is based on the general core microbiomes; we do not show the one for the AMF core microbiomes, as many samples had no or only very few AMF species. The ellipses highlight the differences between the terrestrial (*Cyathea peladensis* (Hieron.) Domin and *Elaphoglossum latifolium* (Sw.) J.Sm.), and epiphytic (*Hymnophyllum polyanthos* (Sw.) Sw., and *Melpomene wolfii* (Hieron.) A.R.Sm. & R.C.Moran) species.

The PERMANOVA test revealed differences in fungal community composition between fern species, with a slight influence of the fertilization treatment used (Table 2). However, the NMDS ordination only displayed a clear separation between epiphytic and terrestrial species, without further differentiation by fern species or treatment (Figure 4).

**TABLE 2:** Results of the permutational analysis of variance comparing the significance of different factors in the relative abundance of the fungi associated to four fern species.

Factors	df	SS	*r* ^2^	*F*	*p*
Treatment	3	1.621	0.02683	1.1412	<.05*
Fern species	3	2.762	0.04571	1.9443	<.001***
Treatment * Fern species	9	4.332	0.07169	1.0165	>.05
Residuals	107	50.665	0.83847		
Total	124	60.426	1.0		

*Note*: Asterisks indicate the levels of statistical significance.

Abbreviations: df, degrees of freedom; *F*, *F* value by permutation; MS, mean of squares; *p*, *p* values; SS, sum of squares.

**FIGURE 5:**
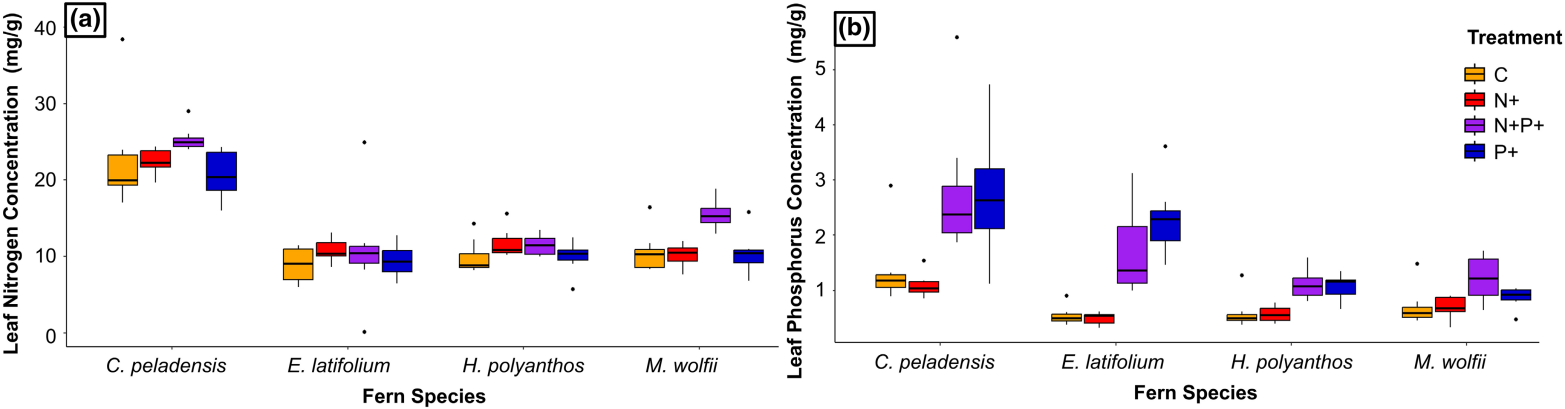
Concentration of nitrogen and phosphorus on the leaves of four fern species (*Cyathea peladensis* (Hieron.) Domin, *Elaphoglossum latifolium* (Sw.) J.Sm., *Hymnophyllum polyanthos* (Sw.) Sw. and *Melpomene wolfii* (Hieron.) A.R.Sm. & R.C.Moran) growing under four nutrient treatments: C+ (control), N+ (nitrogen addition), P+ (phosphorus addition), and N+P+ (nitrogen and phosphorus addition). ANOVA results indicate significative differences in nutrient concentration among treatments by plant species. A: *M. wolfii* (*F* = 7.976, *p* < .001***). B: *C. peladensis* (*F* = 6.303, *p* < .01**), *E. latifolium* (*F* = 6.331, *p* < .001***), *H. polyanthos* (*F* = 0.694, *p* < .001***), and *M. wolfii* (*F* = 4.036, *p* < 0.05*).

Finally, the leaf nutrient analyses showed a significant effect of treatments within species. The leaf phosphorus concentration was significantly higher in treatments P+ and N+P+ compared to C and N+. *Cyathea peladensis* and *E. latifolium* revealed higher P concentrations than the two epiphytic species (Figure 5b, Table 1). Moreover, there were no significant differences relative to the nutrient addition treatment when we focused on the leaf nitrogen concentration, except in the case of *Melpomene wolfii* where treatment N+P+ differed from the others (*F* = 7.976, *p* < .001***) (Figure 5a, Table 1). *Cyathea peladensis* displayed greater leaf N concentration than the other three species.

## 4 DISCUSSION

This is the second study to analyze the diversity of root‐associated fungi in tropical fern species using molecular tools, with Cook and Taylor (2023) sampling roots of two epiphytic fern genera in Costa Rica. Unlike the previous study, we also compared terrestrial and epiphytic species, and explored the effects of 15 years of nutrient addition on the fungal assemblages. Summarizing our main results, we found 1382 ASVs in the core mycobiomes of the roots of the 125 fern individuals studied. There were marked differences in fungal community composition between terrestrial and epiphytic species, with the latter showing a very low presence of arbuscular mycorrhizal fungi (AMF). However, although the two terrestrial and the two epiphytic species belonged to phylogenetically remote fern orders, we did not find differences in AMF community composition between them. Most importantly, we found no direct effect of fertilization on the fungal assemblages, whereas leaf phosphorus concentration was directly influenced by phosphorus fertilization in all the species.

Results that do not support the established hypotheses always receive extra attention to ensure that they are not caused by unsuitable methodological approaches. In our case, we trust the negative results for three reasons. First, in another fertilization study on ferns, using the same metabarcoding approach, we obtained clear differences in the fungal communities between nutrient treatments (Guillen‐Otero et al., 2024), showing that the methods are suitable for detecting responses of root‐associated fungi in ferns. Second, our analyses revealed clear and biologically meaningful differences between fern species and life forms, both for root‐associated fungi and leaf nutrients, again confirming the suitability of the methods. Third, studies of angiosperms on the same study plots obtained clear differences related to the fertilization treatments, both in mycorrhizal associations and in leaf nutrients (Cárate‐Tandalla et al., 2018; Duenas et al., 2020; Homeier et al., 2012; Velescu et al., 2021), showing that the treatments have indeed influenced the fungi and plants in the plots. For all these reasons, we consider that our analyses are sound and robust, and that the lack of a signal of fertilization is a biologically meaningful result of our study. Finally, although this is a standard method to study fungal root associates, we must also consider that our sequencing approach may be selectively bias toward certain species and genera (Berruti et al., 2017; Gardes & Bruns, 1993; Manter & Vivanco, 2007).

Until recent years, studies of mycorrhizal associations in ferns were limited to visual inspections of the root, solely confirming the existence of an association (or lack thereof) and identifying the major fungal group involved (e.g., Field et al., 2015; Kovács et al., 2007; Rimington et al., 2015; Sandoz et al., 2020; West et al., 2009). During the last decade, genomic approaches have allowed full screening of root‐linked fungal assemblages, but studies in fern remain scarce, generally restricted to few species with limited sample sizes, and often using primers specific for arbuscular mycorrhizal fungi (AMF), thus ignoring other fungi (e.g., Cook & Taylor, 2023; Perez‐Lamarque et al., 2022; Sandoz et al., 2020; West et al., 2009). In this study, we sampled 30–32 individuals per species and used a primer combination that allows for the identification of fungi from all major fungal phyla (Guillen‐Otero et al., 2023). This is relevant because dark‐septate endophytes of the Ascomycota are commonly present in fern roots (Lehnert et al., 2017). Their role is poorly understood, but in angiosperms, they have been shown to act as mycorrhizal partners (Jumpponen, 2001; Lukešová et al., 2015). We restricted ASVs representing at least 1% of all reads in a given specimen (what we denominated core microbiome), because the common species are more likely to be functionally relevant (Neu et al., 2021).

Even with this restriction, we found a relatively high diversity of root‐associated fungi, with a total of 1389 ASVs recorded. Most ASVs were only found in a single fern individual and the average number of ASVs per plant was rather low at 15 ASVs per plant. Even the most frequent ASV was only recorded in 35 fern individuals. As a result of this, fungal diversity was largely partitioned between individuals, with on average only 4% of the total fungal diversity found in each single plant. These results might suggest a low specificity of fungi in the studied species, assuming that the specificity of a fern towards certain fungal taxa would be reflected in the differences in fungal communities between fern species as well as higher overlap in the fungi within each species. This interpretation may be reinforced by our inability to detect indicator species for fern species or treatments. This is also concordant with a previous study of epiphytic members of the fern genera *Elaphoglossum* and *Hymenophyllum* in Costa Rica (Cook & Taylor, 2023).

Focusing in more detail on the taxonomic composition of the fungal assemblages, we found that Tremellales (Basiodiomycota) and to a lesser degree Heliotales (Ascomycota) were the dominant orders. Although both orders contain numerous decomposing taxa and their functional role in ferns remains unknown, they also are known to include endophytic fungi (He et al., 2021; Malicka et al., 2022; Taylor et al., 2014). Dark‐septate endophytes (DSE), in particular, are commonly found in fern roots and have been proposed to act as mycorrhizal partners, especially in situations where AMF are lacking, such as in the epiphytic environment (Kessler et al., 2010; Lehnert et al., 2017). It is thus conceivable that at least some of the members of Heliotales and Pleosporales detected by us in the epiphytic specimens also fulfill this role. Among the Glomeromycota (AMF), we found 79 ASVs, with a predominance of families Acaulosporaceae and Glomeraceae coinciding with previous reports for ferns (Guillen‐Otero et al., 2023).

We only observed clear differentiation in AMF community composition between the two terrestrial and the two epiphytic species, and not by applied treatment. This outcome reflects the differences in fungal flora inhabiting these contrasting habitats. AMF were largely absent in the epiphytic samples, which coincides with previous visual inspections of fern roots, both in our study region (Lehnert et al., 2009) and elsewhere (Kessler et al., 2010, 2014; Lehnert et al., 2017). It might reflect the fact that Glomeromycota are soil‐inhabiting fungi and sensitive to habitat disturbances (Brundrett & Tedersoo, 2018; Willis et al., 2013), whereas the epiphytic environment is very dynamic and typically lacks soil, except for an organic layer that sometimes develops on thick branches (Nadkarni et al., 2002).

We did not find any systematic differences in the root‐associated fungi between neither the two terrestrial species *Cyathea peladensis* (Cyatheaceae) and *Elaphoglossum latifolium* (Dryopteridaceae) nor between the two epiphytic ones, *Hymenophyllum polyanthos* (Hymnophyllaceae) and *Melpomene wolfii* (Polypodiaceae). This was unexpected for two reasons: First, Lehnert et al. (2017) proposed that members of fern families that originated and diversified before ATR (Benton et al., 2022), in our case Cyatheaceae and Hymnophyllaceae, would have more specialized mycorrhizal associations as a consequence of their evolution in nutrient‐poor environments. In contrast, members of families that evolved after the ATR would diversity under less nutrient‐limited conditions, thus being less dependent on mycorrhizal partners. Second, Weigand et al. (2022) showed that the abundance of fern species in response to seven years of nutrient addition in our study site differed between fern families, with Dryopteridaceae increasing in abundance and the other three families (Cyatheaceae, Hymenophyllaceae, and the grammitid clade of Polypodiaceae) decreasing. They hypothesized that this might be related to the mycorrhizal specificity of these groups, but although our study did not directly test this hypothesis, the high variability between individuals and the lack of distinction between species, makes this unlikely.

Regarding the effects of the nutrient addition experiment, we found no clear differences in the fungal communities of the four fern species. However, we observed distinct responses of leaf nutrient concentrations to phosphorus addition within species. These results partially contrast with previous analyses of woody plants at the same area, where nutrient addition, especially of phosphorous, led to a decrease AMF presence (Duenas et al., 2020), and an increase in foliar N and P concentrations in most of the studied trees and tree seedlings (Cárate‐Tandalla et al., 2018; Homeier et al., 2012). Although we observed a slight decrease in Glomeromycota presence on the terrestrial plants growing in P fertilized plots, this was not significant. Thus, although a previous study detected shifts in fern species abundances as a response to nutrient addition (Weigand et al., 2022), we did not find parallel changes among the fungal assemblages on fern roots. We thus propose that contrary to the suggestions of Weigand et al. (2022), the shifts in the abundances of fern species in the different treatments of the NUMEX experiment are not linked to mycorrhizae but may rather be due to indirect effects of fertilization, for example, shifts in the growth of both the ferns and the other plants that modify their competitive abilities, and hence lead to changes in their abundances.

## 5 CONCLUSIONS

Our study reveals that the relationships between ferns and their root‐associated fungal communities (arbuscular mycorrhizal fungi communities in particular) are not closely dependent on nutrient availability. Naturally, with only four species analyzed at a single site, generalizations should be made with care. However, our study coincides with a previous greenhouse experiment, in which the degree of mycorrhization in the temperate fern species *Struthiopteris spicant* was found to be strongly influenced by light availability (hence carbon limited), and not by nutrient availability (nutrient limited) (Guillen‐Otero et al., 2024). Although this finding suggests that mycorrhization in ferns is not as linked to nutrient availability as it is in other vascular plant groups, there is still a need for more detailed studies to understand its nature and role in ferns.

## AUTHOR CONTRIBUTIONS

M.K. and J.H. conceived the idea. M.K. designed this study. M.K. and T.G.O. collected the samples. T.G.O. performed the molecular analysis. J.H. carried out the nutrient concentration analysis. T.G.O. and M.K. completed the statistical analysis. M.K. and T.G.O. wrote the manuscript, and all authors contributed substantially to the revisions.

## FUNDING INFORMATION

This study was entirely supported by the Swiss National Science Foundation (project: 188498) and the Georges and Antoine Claraz‐Schenkung.

## CONFLICT OF INTEREST STATEMENT

The authors declare no competing interests.

## Supporting information


Figure S1.

**Figure S2.**.

Table S1.
Table S2.

Appendix S1:

## Data Availability

All the sequence data obtained during this study have been deposited in the National Center for Biotechnology Information (NCBI) Sequence Read Archive (SRA) database with BioProject ID PRJNA1033591 (Temporary reviewer link: http://www.ncbi.nlm.nih.gov/bioproject/1033591). All datasets supporting the conclusions of this article are included within the article and its supporting information.
