# Supplementary figures and images for: Fern mycorrhizae do not respond to fertilization in a tropical montane forest
Source: Plant Environ Interact. 2024 Mar 29;5(2):e10139. doi: 10.1002/pei3.10139 (PMC10979390; doi:10.1002/pei3.10139)

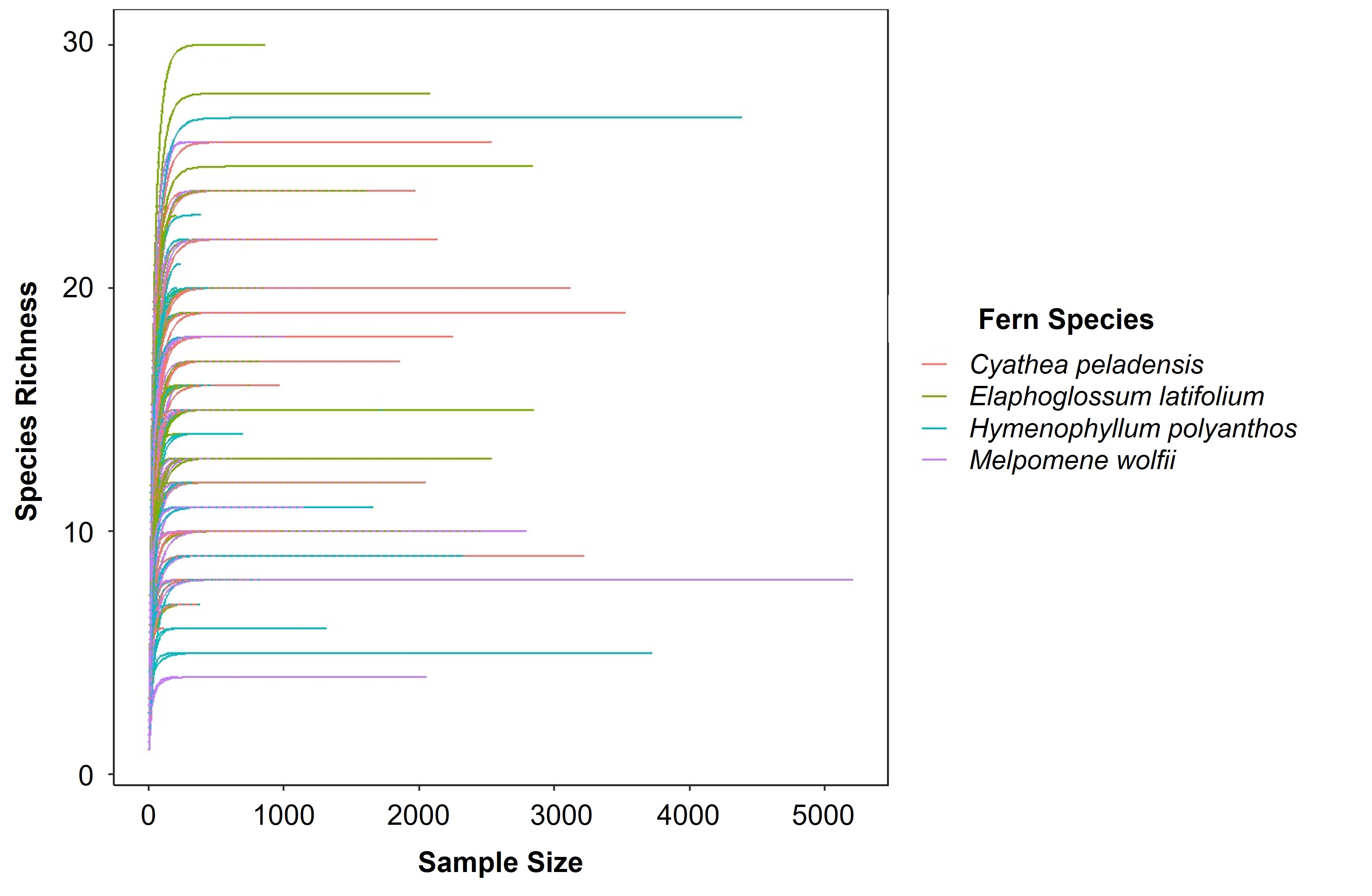

Supplement: Supplementary file 1 — Figure S1. [file PEI3-5-e10139-s001.jpg]

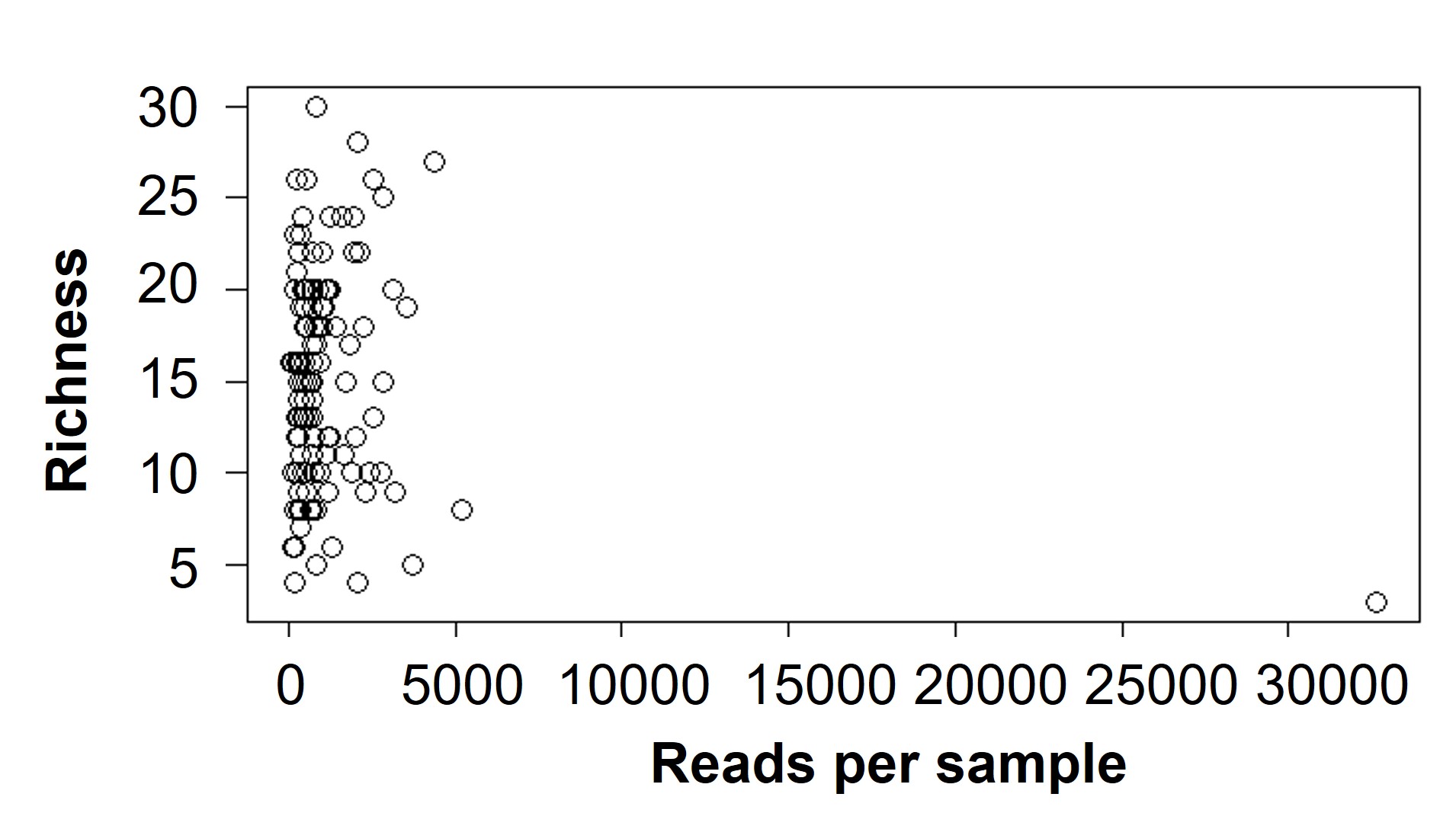

Supplement: Supplementary file 2 — Figure S2.. [file PEI3-5-e10139-s002.jpg]
