# Supplementary material for: Fern mycorrhizae do not respond to fertilization in a tropical montane forest
Source: Plant Environ Interact. 2024 Mar 29;5(2):e10139. doi: 10.1002/pei3.10139 (PMC10979390; doi:10.1002/pei3.10139)
Supplement: Supplementary file 3 — Table S1. Table S2. [file PEI3-5-e10139-s004.docx]

Table 1. Diversity partitioning data for the general fungal core microbiome.

| **Species** | **α1** | **%** | **α2** | **%** | **B1** | **%** | **B2** | **%** | **γ** |
| --- | --- | --- | --- | --- | --- | --- | --- | --- | --- |
| Cyathea peladensis | 15.20 | 3.33 | 114 | 32.29 | 98.80 | 21.67 | 342 | 75.00 | 456 |
| Elaphoglossum latifolium | 16.88 | 3.13 | 130 | 24.07 | 113.13 | 20.95 | 410 | 75.93 | 540 |
| Hymenophyllum polyanthos | 13.47 | 3.13 | 107.75 | 25.00 | 94.28 | 21.88 | 323.25 | 75.00 | 431 |
| Melpomene wolfii | 14.00 | 3.23 | 113.25 | 26.09 | 99.25 | 22.87 | 320.75 | 73.91 | 434 |

Table 2. Diversity partitioning data for the arbuscular mycorrhizal fungi core microbiome.

| **Species** | **α1** | **%** | **α2** | **%** | **B1** | **%** | **B2** | **%** | **γ** |
| --- | --- | --- | --- | --- | --- | --- | --- | --- | --- |
| Cyathea peladensis | 1.97 | 3.33 | 14.75 | 25 | 12.78 | 21.67 | 44.25 | 75 | 59 |
| Elaphoglossum latifolium | 0.63 | 3.13 | 5 | 25 | 4.38 | 21.88 | 15 | 75 | 20 |
| Hymenophyllum polyanthos | 0.09 | 3.13 | 0.75 | 25 | 0.66 | 21.88 | 2.25 | 75 | 3 |
| Melpomene wolfii | 0.33 | 3.33 | 2.5 | 25 | 2.17 | 21.67 | 7.5 | 75 | 10 |
